# Energetic and kinetic dataset on interaction of the vacancy and self-interstitial atom with the grain boundary in α-iron

**DOI:** 10.1016/j.dib.2016.03.052

**Published:** 2016-03-19

**Authors:** Xiangyan Li, Wei Liu, Yichun Xu, C.S. Liu, B.C. Pan, Yunfeng Liang, Q.F. Fang, Jun-Ling Chen, G.-N. Luo, Guang-Hong Lu, Zhiguang Wang

**Affiliations:** a Key Laboratory of Materials Physics, Institute of Solid State Physics, Chinese Academy of Sciences, P.O. Box 1129, Hefei 230031, PR China; b Hefei National Laboratory for Physical Sciences at Microscale and Department of Physics, University of Science and Technology of China, Hefei 230026, PR China; c Environment and Resource System Engineering, Kyoto University, Kyoto 615-8540, Japan; d Institute of Plasma Physics, Chinese Academy of Sciences, Hefei 230031, PR China; e Department of Physics, Beihang University, Beijing 100191, PR China; f Institute of Modern Physics, Chinese Academy of Sciences, Lanzhou 730000, PR China

## Abstract

We provide the dataset of the vacancy (interstitial) formation energy, segregation energy, diffusion barrier, vacancy-interstitial annihilation barrier near the grain boundary (GB) in bcc-iron and also the corresponding interactive range. The vacancy-interstitial annihilation mechanisms in the bulk, near the GB and at the GB at across scales were given.

**Specifications Table**

**Table:** 

Subject area	*Physics*
More specific subject area	*Materials science*
Type of data	*Table, figure, movie*
How data was acquired	*Molecular dynamics simulations, molecular statics calculations and the object kinetic Monte Carlo simulations*
Data format	*Raw, analyzed*
Experimental factors	*Interaction of the vacancy and interstitial with the GB at across scales was investigated*
Experimental features	*Theoretical simulations at across scales*
Data source location	*Center for Computation Science, Hefei Institutes of Physical Sciences, Hefei, PR China*
Data accessibility	*Data is with this article*

**Value of the data**

• Increase our knowledge of radiation-produced point defects behaviors near the GB at across scales.
• Provide insights into the annihilation mechanism of the vacancy-interstitial in the bulk and near the GB.
• Provide energetic and kinetic parameters describing interaction of the vacancy, interstitial with the GB and also interactive range parameters.
• The interaction parameters at atomic scales are very useful for any further investigation of radiation response of the GB by using high level of coarse-grained simulation techniques.

## 1 Data

The shared data includes Fig. 1, Fig. 2, Fig. 3, Fig. 4, Fig. 5, Fig. 6, Fig. 7, Fig. 8, Fig. 9, Fig. 10, Fig. 11, Fig. 12, Fig. 13, Fig. 14, Fig. 15, Fig. 16, Fig. 17, Fig. 18, Fig. 19, Table 1, Table 2 associated with molecular dynamics (MD) simulation of the primary damage near the GB, molecular statics (MS) calculation of the SIA-V interaction near the GB and object Kinetic Monte Carlo (OKMC) investigation of the dynamic interaction of the SIA-V pair near the GB. Movie S1 is included as supplementary material to this article.

Supplementary material related to this article can be found online at doi:10.1016/j.dib.2016.03.052.

The following is the Supplementary material related to this article Movie S1.

Movie S1 Illustration of diffusion and annihilation of *SIA*s and vacancies near the GB. Here the *SIA* is represented by one green sphere. The red filled ellipse surrounded the *SIA* represent the annihilation region with low energy barriers. The *V* is represented by one cubic box. Symbols *SIA1*, *SIA2* and *SIA3* mark three single *SIA*s, while *V1*, *V2* and *V3* are for three single vacancies. The exact GB position is indicated by one black line. The shadow near the line represents the GB-enhanced diffusion region where *SIA*s and vacancies have small diffusion energy barriers. The physical picture presented in the movie is from molecular dynamics simulations, molecular statics calculations and object kinetic Monte Carlo investigation of interaction among radiation-created *SIA*s, vacancies and the GB.

## 2 Experimental design, materials and methods

### 2.1 Experimental design

Molecular dynamics simulations of the primary radiation damage were firstly performed to reveal possible atomic evolution processes of the vacancy and self-interstitial atom near the GB. Then these processes were parameterized by calculating vacancy/interstitial formation energy, diffusion energy barrier and the annihilation energy barrier with the molecular statics method. Finally, the obtained parameterized processes were considered as the input of the object-kinetic Monte Carlo method to reveal the macroscopic evolution of the vacancy and the interstitial.

#### 2.1.1 GB models and atomic potential

The GB studied in this work is a Ʃ5 (3 1 0)/[0 0 1] symmetric tilt GB in bcc iron, where Σ indicates the degree of geometrical coincidence at the GB, (3 1 0) denotes the plane of the GB and [0 0 1] denotes the rotation axes. The creation and relaxation procedures are identical to the procedures described in our previous work [4], [6], [7]. Periodic boundary conditions were applied in the two directions parallel to the GB plane, but the fixed boundary condition was selected for the direction normal to the GB plane. The simulation cells consist of a moving region sandwiched between two rigid regions, similar to simulation cells used in other studies [4], [5], [6], [7]. The GB energy was minimized through the relaxation of all non-rigid atoms and the rigid-body translations of one grain relative to the other in all three Cartesian directions at 0 K. The lowest energy of the relaxed GB structure was calculated to 0.99 J/m^2^. Six other GBs, i.e., Σ5 (2 1 0), Σ13 (5 1 0), Σ17 (4 1 0), Σ25 (7 1 0), Σ29 (5 2 0) and Σ37 (6 1 0), were constructed and relaxed analogously.

The inter-atomic potential proposed by Mendelevet al. as part of their embedded-atom-method [8] (Potential 2 in Ref. [8]) was used to model the inter-atomic interaction. At the short range of 1.0 Å the potential has been splined to the universal high-energy empirical potential established by Ziegler *et al.* [9]. The obtained potential was then applied to study the primary radiation damage near the GBs in iron. Tests showed that this potential correctly predicted the properties of the point defects. The bulk *V* formation energy was calculated to 1.72 eV, and the diffusion barrier to 0.63 eV. The stable configuration of a single *SIA* was identified as the 〈1 1 0〉 dumbbell configuration with a formation energy of 3.53 eV and a diffusion energy barrier of 0.33 eV. The potential was therefore proved suitable for investigating the diffusion and annihilation of *V*s, *SIA*s and corresponding *V*-*SIA* pairs.

For the MD simulations, a large GB model with a size of about 180×70×90 Å^3^ was constructed containing 95,520 atoms. The atoms in the outermost layers of the moving region, with a thickness two times the lattice constant, were coupled with a velocity-rescaling thermostat to absorb the cascade energy and maintain the system temperature at the chosen value. The model is illustrated in Fig. 1(a) in Ref. [1]. The size of the simulation cell was carefully chosen to avoid interactions between two cascades in the two neighboring cells. This was confirmed by the visualization of the MD simulations. The size of the model in the direction normal to the GB was then adjusted to avoid interactions between the fixed surface and the GB.

The thermodynamic and kinetic properties of the relevant processes identified in the MD simulations were then calculated by MS simulations using a smaller GB model. This model consisted of 3792 atoms and had a size of about 73×27×23 Å^3^. The ground state for the GB core structure is illustrated in Fig. 1(b) in Ref. [1]. We have first checked whether the model size was large enough for calculating the energetic and kinetic properties of *SIA*s and *V*s and for calculating the energy landscapes of the *SIA*-*V* pair near the GB.

For the OKMC simulations, a square-shaped grain model was built with periodic boundary conditions imposed in all three dimensions, as illustrated in Fig. 1(c) in Ref. [1]. The GB was placed at the center of the cell. The physical picture and related approximations underling the model are shown in Fig. 1(d−f) in Ref. [1]. In the bulk and at the GB, the GB model structure is periodic three-dimensionally and two-dimensionally, respectively. Nevertheless, the lattice sites or the defect sites within a structural unit are often non-equivalent and the local atomic environment therein is complex. This may lead to existence of multiple meta-stable sites for the defect location (referred to the MS calculation results in the text). As a result, as a defect moves in the real coordinate space, it not only visits stable states but also visits meta-stable ones. For example, for a vacancy diffusing in the bulk, there exists a meta-stable site at the center of the path from the vacancy to its first-nearest neighbors. Near the GB, the system loses its structural periodicity as it transits from one grain to the other. Although the GB generally acts as the defect sink, the exact trapping process may involve several sequential transitions from the bulk region to the GB. Correspondingly, the real energy landscapes for the defect near the GB and at the GB and even in the bulk were found to be rough and rugged. The nature of the OKMC simulation of defects behaviors is to use a motion rate to model the local potential field. Emergence of lots of transitions related to these meta-stable states will severely reduce the computation efficiency of the OKMC if all the transitions are considered into the model. Therefore, in the present coarse-grained OKMC modeling of defect evolution, only the transitions from one stable site to another were considered and the transitions between a stable state and a meta-stable one or between two meta-stable states were omitted.

The real energy landscape for the *V* and *SIA* near the GB was approximated by an squared potential well (Fig. 1(e) in Ref. [1]). The width of the well was defined as the interactive range of the defect with the GB. The depth of the well is the energy reduction as the defect segregates into the GB from the bulk. This approximation was believed to have little effect on the evolution of the defect (binding energy), since the interactive range of the defect with the GB was calculated to be small than 1.0 nm [30] and the events near the GB was estimated to be very fast due to GB-enhanced diffusion or annihilation of the defect compared with other events in the bulk or at the GB. Particularly this is a good approximation for the *SIA* due to the spontaneous absorption of the *SIA* near the GB (referred to the MS calculation results in the text and also Ref. [6]).

In the simulation, the low-barrier problem was also encountered. As the system evolved, the system was found to occasionally fall into a region of the landscape where a series of states exist. The transitions between these states only overcome a low barrier, leading to a large transition rate and consequently frequent visiting of these states, e.g., the vacancy migration from one stable site on one GB side to the symmetric site on the other GB side nearly barrier-freely. As a result, the timescale that OKMC can access is severely shortened. In this case, multiple states were treated as a super-basin (Fig. 1(f) in Ref. [1]). By adopting this approximation, the time for the defect to wander at a meta-stable state was set to be zero, in other words, accelerating the overall transition rate from one stable state to another. Consequently, the corresponding timescale was underestimated.

Above approximations lead to the following pictures. In the bulk, the *V* and *SIA* undergo three dimensional walk with migration rates *r*_1_, *r*_2_, *r*_3_, *r*_4_, *r*_5_, *r*_6_, *r*_7_, *r*_8_ along eight directions in a body-centered cubic cell (Fig. 1(d) in Ref. [1]). These rates are equal independent of the motion direction. At the GB, the *V* and *SIA* move two dimensionally with rates *r*_1_, *r*_2_, *r*_3_, *r*_4_ along four directions. Rates *r*_1_=*r*_3_ and *r*_2_=*r*_4_. In the vicinity of the GB, the *V* and *SIA* have two motion degree, segregation into the GB or escape from the GB with the corresponding rates $r_{seg}^{V}$, $r_{seg}^{SIA}$ and $r_{escape}^{V}$,$r_{escape}^{SIA}$. The annihilation rate of the *V*-*SIA* pair depends on the location of the *V* and *SIA*. As both the *V* and *SIA* are in the bulk or at the GB, the annihilation rates are $r_{ann}^{bulk}$ and $r_{ann}^{GB}$, respectively. If one of the *V* and *SIA* is located in the bulk and the other resides at the GB, their annihilation rate is $r_{ann}^{near\;\mathrm{GB}}$.

These event rates for the defect object were calculated by $r=v_{0}\exp(-E_{a}/k_{B}T)$, where $v_{0}$ is the vibration frequency of atoms in iron bulk and is generally assumed to be 10^12^/s. The Boltzmann constant *k*_*B*_ has a value of 8.617×10^−5^ eV/K. *E*_*a*_ and *T* are activation energy and system temperature, respectively. Here, *E*_*a*_ was defined as the overall barrier that the defect overcomes when migrating from one stable site to another. *E*_*a*_ in $r_{escape}^{V}$/$r_{escape}^{SIA}$is the sum of the binding energy and the diffusion energy barrier of the *V*/*SIA*. For the annihilation, *E*_*a*_ was taken to be zero within a certain range of the *V*-*SIA* pair considering spontaneous annihilation. Within the low-barrier region, *E*_*a*_ was approximated to be half the diffusion barrier of the defect that has a smaller migration energy barrier compared with its counterpart. The parameters related to *E*_*a*_ and other interactive range parameters were deduced from MS calculations.

#### 2.1.2 MD simulation of the primary damage near the GB

The velocity-Verlet method was used for solving the motion equations during the MD simulations. A temperature of 1000 K was selected for the activation of the *V* near the GB on the MD simulation time scale and to help us observe the generation and evolution of irradiation defects near the GB.

Before irradiating the system, the GB was firstly relaxed assuming a temperature of 1000 K for 10 pico-seconds (ps) with a time step of 2×10^−3^ ps to reach thermal equilibrium. Then, an atom within about 70 Å of the GB and located at the center of the cross-sectional plane parallel to the GB plane was selected as the primary knock-on atom (PKA). The atom was then given a kinetic energy of 3 keV and a velocity vector directed perpendicularly towards the GB. The PKA induced a collision cascade at the GB, and a smaller time step of 1×10^−4^ ps was used for the next 2 ps to observe the collision cascade. After the cascade cooled down, the simulation was allowed to run for another 10,000 ps with a time step of 2×10^−3^ ps. For each selected distance of the PKA, 12 independent simulation runs were performed.

To characterize the damaged GB structure, we visualized the GB structure and counted the defects in the bulk region, particularly within the GB. In this work, the bulk region in the GB-containing system was defined as those parts of the system with a distance of at least 5 Å to the GB plane. To count the number of irradiation defects, we also employed the Wigner-Seitz cell method [10] to identify the defects.

#### 2.1.3 MS calculation of the SIA-V interaction near the GB

The defect formation energy at the site αis defined as:

(1) $$E_{f}^{\alpha }=E_{GB}^{\alpha }\pm E_{coh}-E_{GB},$$

Where positive sign corresponds to the *V* and a negative sign corresponds to the *SIA. E_coh_* is the cohesive energy per atom for a perfect bcc iron lattice (−4.12 eV); $E_{GB}^{\alpha }$ and $E_{GB}$ denote the total energies of the simulation cell with and without the *V* or *SIA*, respectively. Defect formation energy means the energy cost of creating a defect, which is the thermodynamic description of defect properties. In the MS calculation of the *V*/*SIA* formation energy, when a *V*/*SIA* was created (by removing or adding an atom) in a system, the system was relaxed using the steepest decent method.

To investigate the kinetics and dynamics of the *V* and *SIA* diffusion and annihilation in the bulk and near the GB, we combined the dimer method [3] with NEB method [2]. The NEB method was mainly used to determine the minimum energy path for defect diffusion and annihilation, as well as the corresponding energy barrier. The states that have been relaxed for calculating defects formation energy served as the possible initial and final states of a diffusion or annihilation process. During the NEB calculation, the conventional NEB method does not exactly yield a saddle point. We added dense images along the initial path: 28 intermediate images were inserted between the initial and final states using the linear interpolation method.

The dimer method was mainly used to explore the *SIA*-*V* annihilation mechanisms in the bulk and near the GB rather than fully explore the potential energy surface of the system of interest. Once the transition was detected, the structural variation during the transition was analyzed. Only the transitions corresponding to *SIA*-*V* annihilation was further analyzed to reveal possible annihilation mechanisms. In the calculations, the dimer method was firstly used to find the approximate minimum energy path, which was then rigorously relaxed by applying the NEB method. In the modified dimer method [3], we used curvatures at rotation angles of 0°, 45° and 90° to calculate the Fourier coefficients *a*_0_, *a*_1_ and *a*_2_. Thereby we were able to calculate the curvature with high accuracy. In addition, we only activated the atoms within a certain range of the event region. The center for the activated region can be at the site of the defect introduced. The radius for the activated region was found to vary from 3 to 8 Å, which makes the dimer search well converge. A choice of a large radius makes the dimension of the potential energy surface too large to be explored and the search was therefore difficult to converge. The un-relaxed system with defects was found to be a good initial configuration for dimer searches. The dimer separation was set to 0.05 Å. The critical angle for the dimer rotation was 0.01 rad. The step for translations of the dimer was 0.1 Å. For each dimer search, the initial dimer direction was randomly given by assigning a unit random vector or by assigning a specific direction, e.g., the direction of the *V* migration. For a given initial state and activation direction, 20 independent dimer searches were conducted.

#### 2.1.4 OKMC investigation of the dynamic interaction of the SIA-V pair near the GB

Two different **OKMC** models were used and two typical temperatures, 200 and 300 K, were selected. At 200 K, the *SIA* in the bulk region became activated whereas the *V* remained immobile. At 300 K, both the *SIA* and the *V* became activated. In the first model, only an *SIA* or a *V* segregation event was included in the model. This model was employed to test whether the GB acts as an effective sink for radiation-defects. The grain size was chosen to 15, 50, 100, and 700 nm. The experimental values previously reported for the average grain size (49, 96 and 700 nm) served as reference [11]. The initial *SIA*/*V* concentration was set to 30 appm in the GB system with a grain size of 15 nm. The concentration was only 1 appm for the systems with a grain size of 50 and 100 nm, and 0.1 appm for the system with a grain size of 700 nm. The choice of the defect concentration was based on two considerations, i.e., the statistics of the results and the computational cost (the combination of a high defect concentration with a large grain size were beyond the capabilities of the OKMC simulation).

In the second model, both segregation and annihilation processes were considered, including the diffusion of the *SIA* and the *V* and their annihilation in the bulk region, near the GB and at the GB. The model was used to investigate the segregation and annihilation behavior of *SIA*s and *V*s over long timescales, especially the coupling of the segregation and the annihilation processes. The grain size was selected to a typical value of 50 nm. For comparison, the corresponding *SIA*-*V* pair concentration was chosen to 10 and 100 appm. In both models, only point defects and single *SIA*s and *V*s, were considered.

## Figures and Tables

**Fig. 1:**
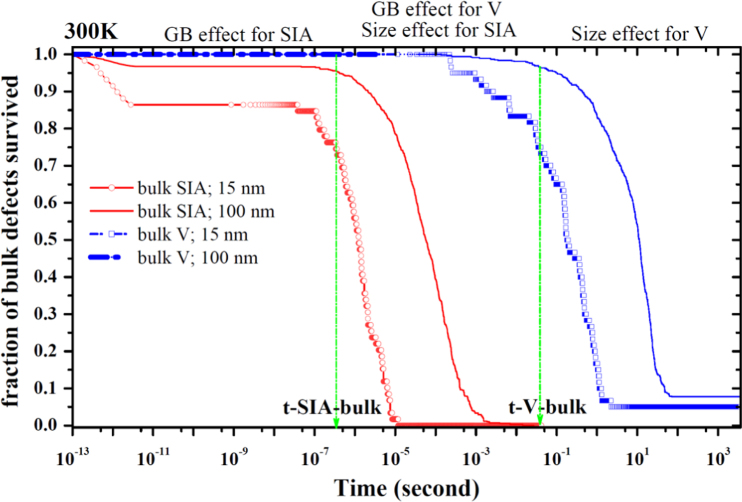
Evolution of the fraction of the vacancy (*V*) and self-interstitial atom (*SIA*) survived in the bulk region with time at room temperature. Here *t-SIA-bulk* and *t-V-bulk* denote the time for the *SIA* and *V* to jump one step in the bulk at 300 K, respectively. The initial concentration of the *SIA* (*V*) is 30 appm in the grain boundary (GB) model with the grain size of 15 nm. In the GB model with the grain size of 100 nm, the concentration is 1 appm.

**Fig. 2:**
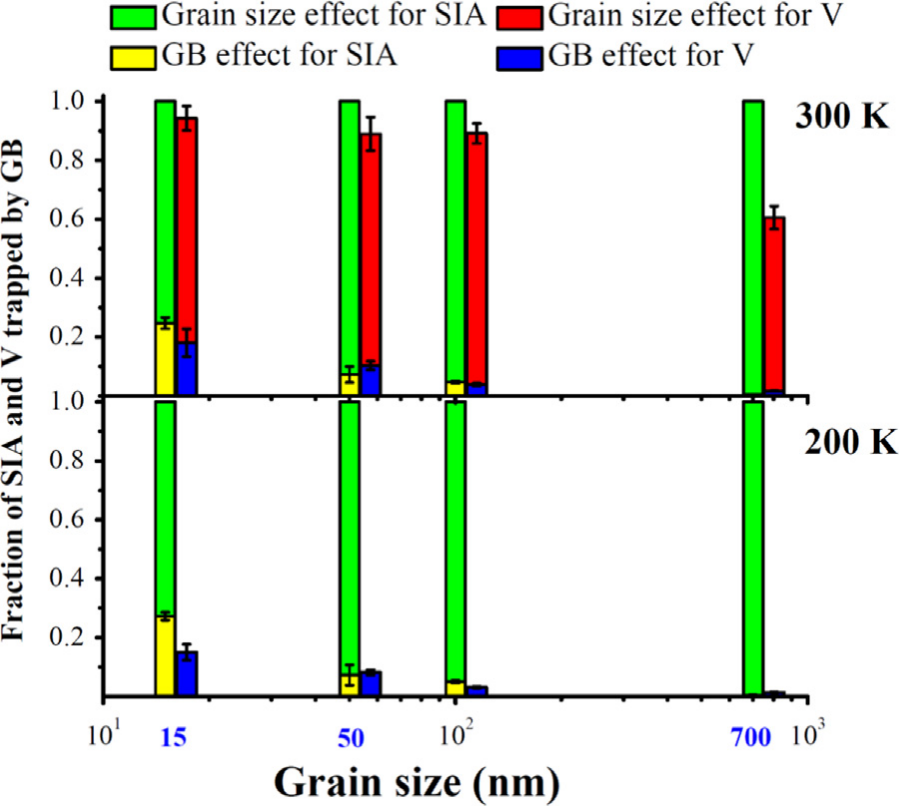
Fraction of the *SIA* and *V* trapped by the GB as a function of the grain size at 300 K and 200 K. The two types of effects, the GB effect and the grain size effect, are shown and defined in Fig. 1.

**Fig. 3:**
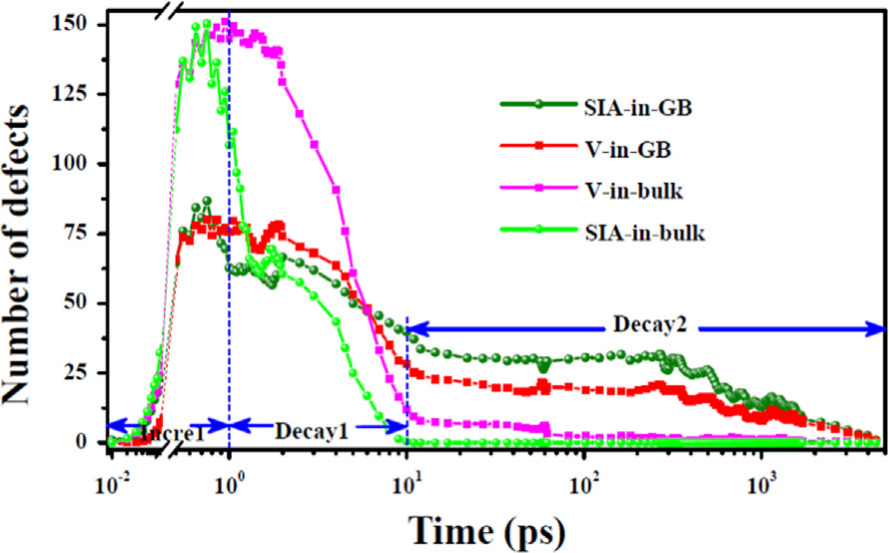
The evolution of interstitial (vacancy) number with time in the grain interior and boundary at 1000 K. Here, *Incre1*, *Decay1* and *Decay2* indicate three stages of defect number evolution. The GB corresponds to the system in Fig. 2(b) in Ref. [1].

**Fig. 4:**
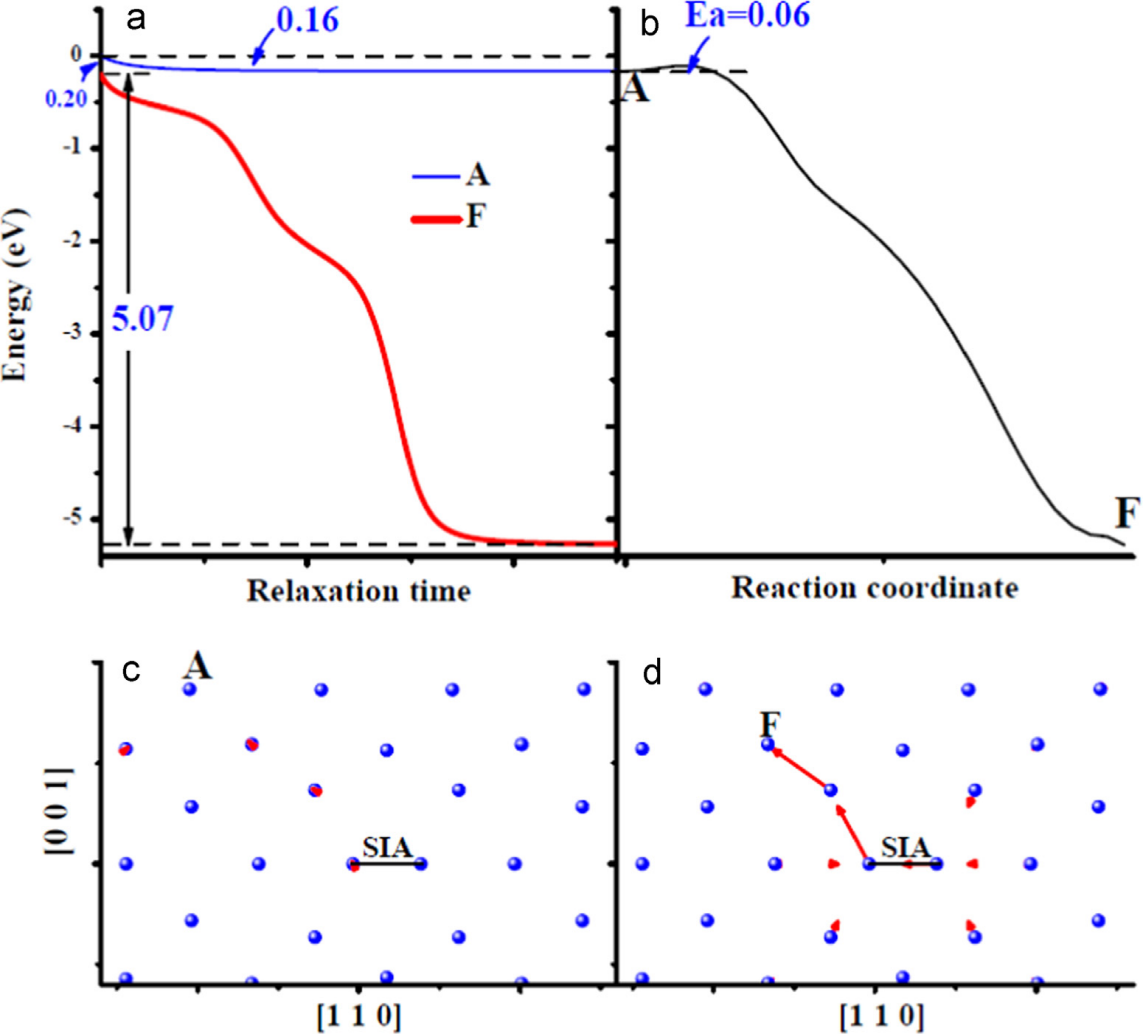
Comparison of an spontaneous *V*-*SIA* annihilation process with an activated one in the bulk. (a) shows the energy variations of the system with the relaxation time with one *V* respectively at sites *A* and *F* (their locations are shown in (c) and (d)) near one *SIA*. The energy of the system with one *V* created at site *A* without relaxation has been chosen as an energy reference. (b) shows the energy variation along the path from *A* to *F* obtained from one nudged-elastic-band (NEB) [2] calculation. The relaxed system with one *V* at site *A* and *F* has been selected as the initial and final states, respectively. The symbol *E*_*a*_ is for the activation energy barrier. (c) and (d) show the structural relaxation after introducing one *V* at sites *A* and *F*, respectively. The position of an *SIA* is marked by one black line along [1 1 0]. In (c) and (d), the structural relaxation is visualized by joining the red arrow from the initial to final location of each atom involved.

**Fig. 5:**
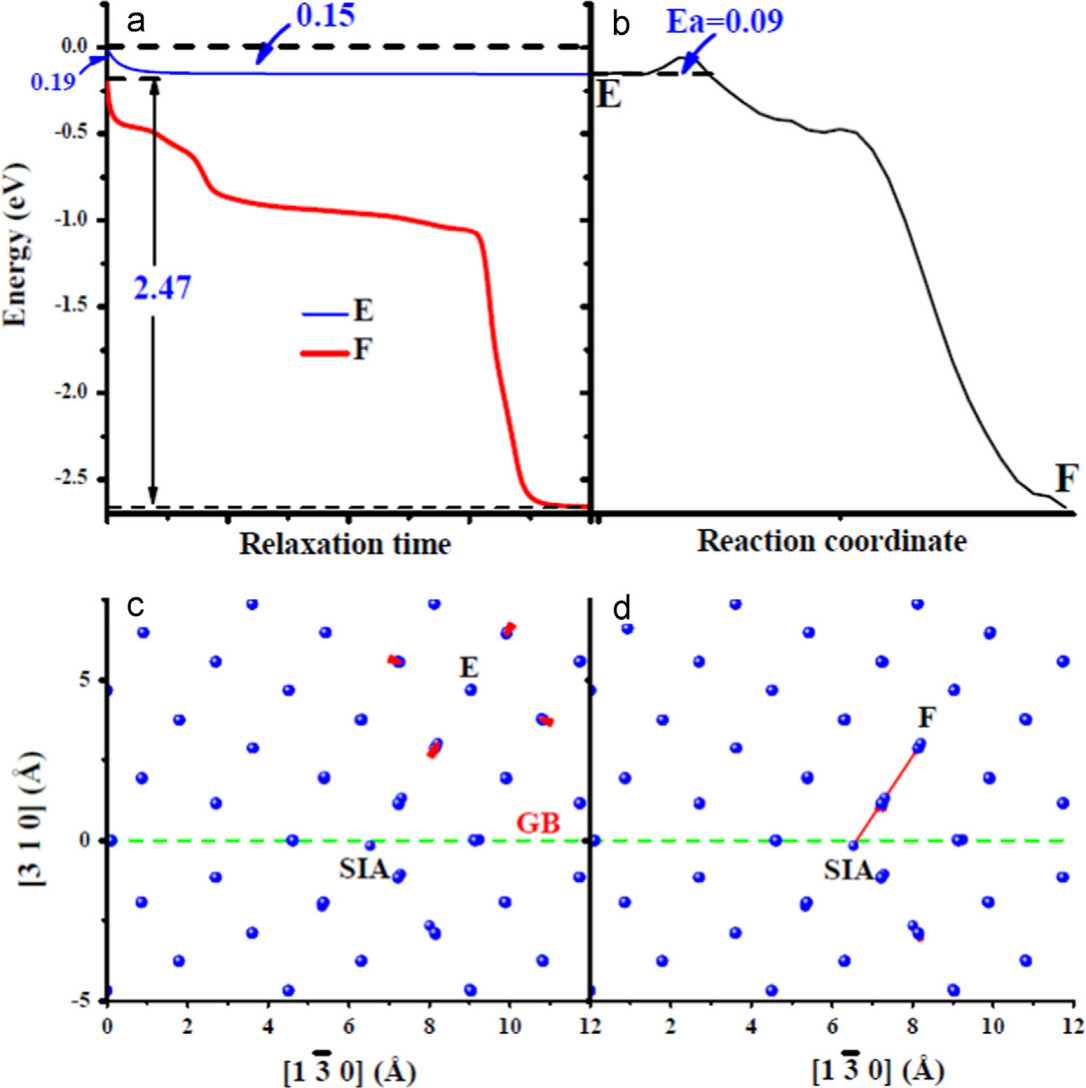
Comparison of an spontaneous V-SIA annihilation process with an activated one near the GB. (a) shows the energy variations of the system with the relaxation time with one *V* respectively at sites *E* and *F* (their locations are shown in (c) and (d)) near one *SIA*. The energy of the system with one *V* created at site *E* without relaxation has been chosen as an energy reference. (b) shows the energy variation along the path from *E* to *F*. The relaxed system with one *V* at site *E* and *F* has been selected as the initial and final states, respectively. (c) and (d) show the structural relaxation after introducing one *V* at sites *E* and *F*, respectively. The visualization method is identical to that in Fig. 4(c) and (d). In (c) and (d), the dashed green line indicates the GB position.

**Fig. 6:**
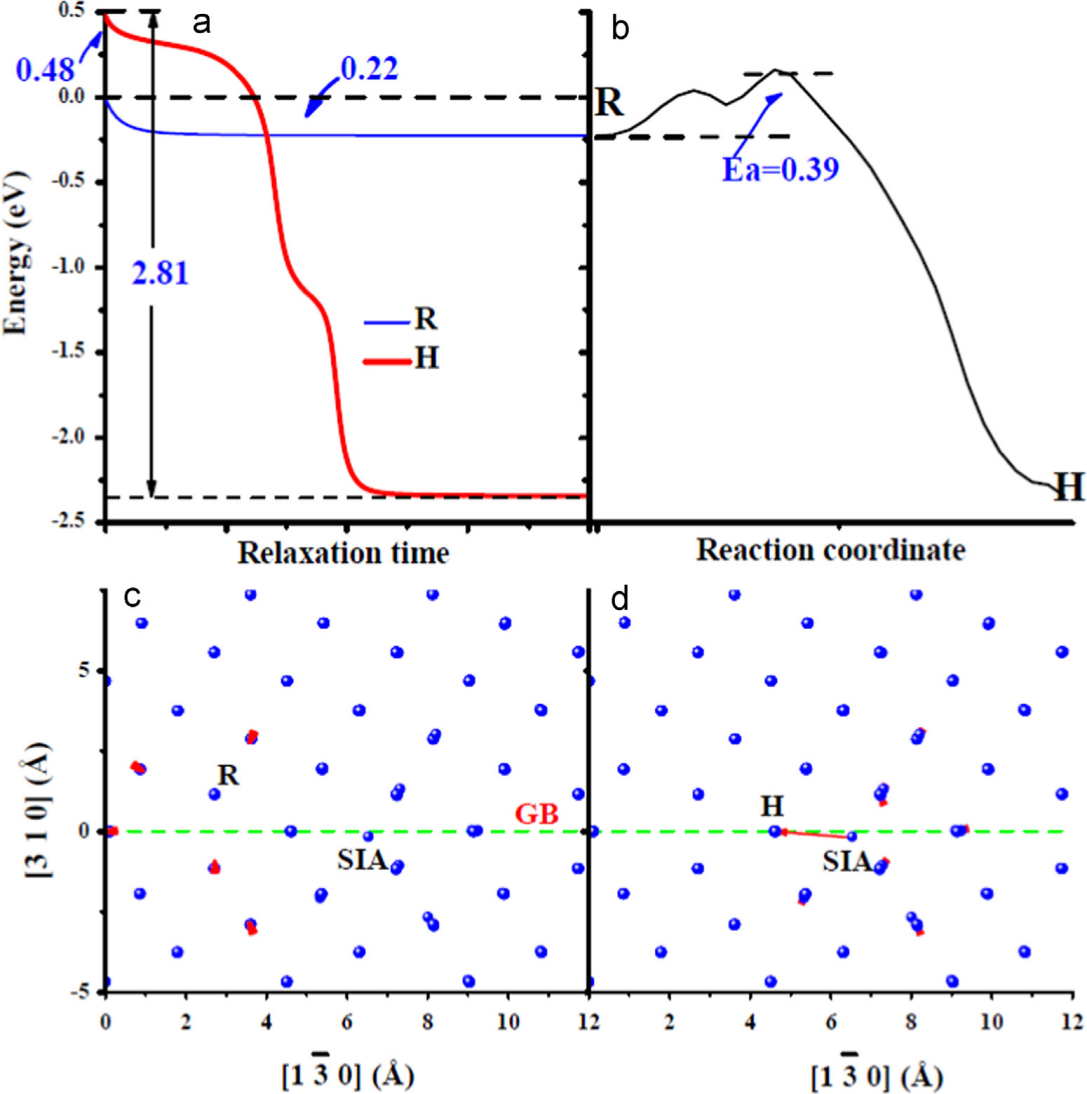
Comparison of an spontaneous *V-SIA* annihilation process with an activated one at the GB. (a) shows the energy variations of the system with the relaxation time with one *V* respectively at sites R and H (their locations are shown in (c) and (d)) near one *SIA*. The energy of the system with one *V* created at site R without relaxation has been chosen as an energy reference. (b) shows the energy variation along the path from R to H. The relaxed system with one *V* at site R and H has been selected as the initial and final states, respectively. (c) and (d) show the structural relaxation after introducing one *V* at sites R and H, respectively. The visualization method is identical to that in Fig. 4(c) and (d). In (c) and (d), the dashed green line indicates the GB position. Site R is one of most stable position for the *V* at the GB.

**Fig. 7:**
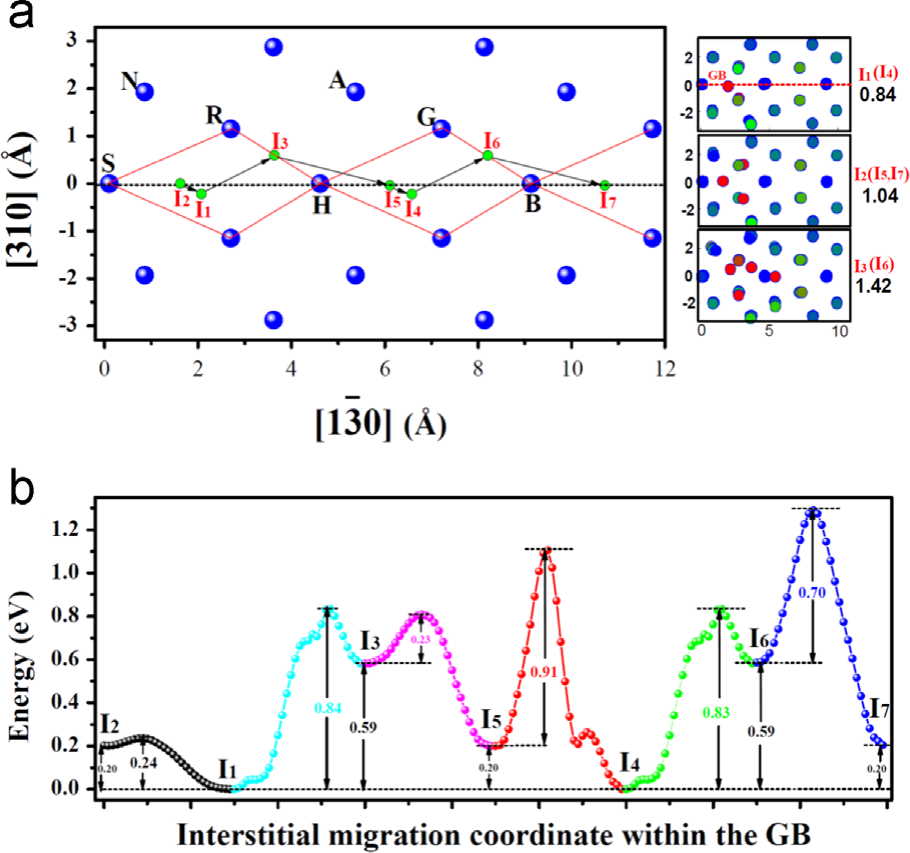
Interstitial diffusion along axis [1 $\overset{¯}{3}$ 0] within the GB (a) and the corresponding energy profiles on the path (b). In (a), the larger blue spheres represent lattice atoms, while the smaller green spheres represent *SIA* sites *I*_1_−*I*_7_ at the GB. The black dashed horizontal line indicates the boundary position. The red lines mark the structural individual at the GB. The black arrows show the corresponding diffusion paths. A perfect period covers from *S* to *B* along [1 3̄ 0]. The configurations for *I*_1_−*I*_7_ are shown on the right of the figure. *I*_1_ and *I*_4_ are the stable sites for the *SIA* with formation energy of 0.84 eV, while *I*_2_, *I*_5_, and *I*_7_ meta-stable ones with energy of 1.04 eV. The high energy meta-stable sites *I*_3_ and *I*_6_ have energy of 1.42 eV. In (b), the energy of the system with one *SIA* at the stable site is chosen as reference.

**Fig. 8:**
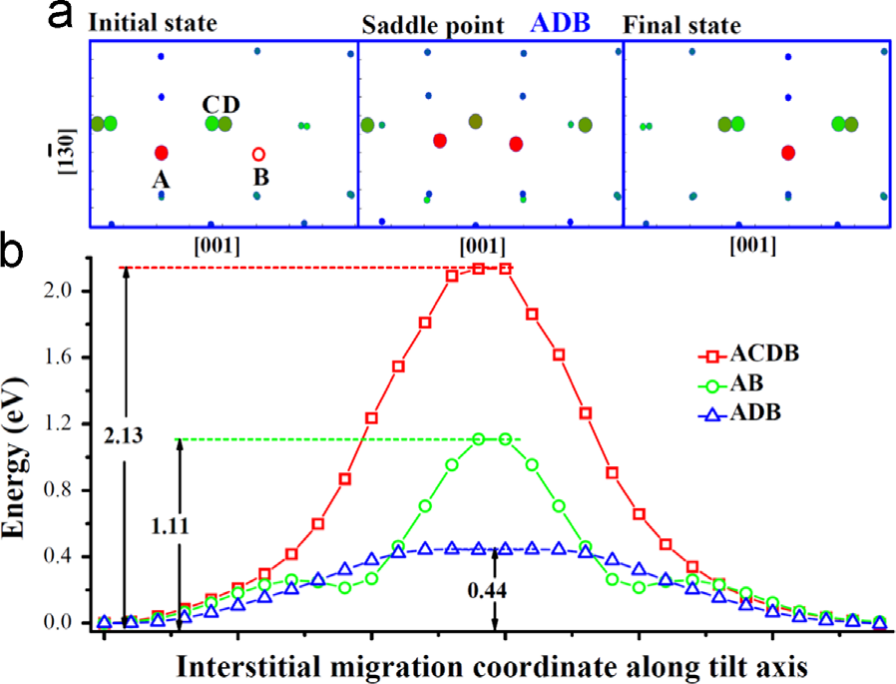
Interstitial diffusion along axis [0 0 1] within the GB. In (a), the configurations for the two neighboring stable *SIA* sites and the saddle point configuration for the transition *ADB* are given. Here the bigger spheres represent the *SIA* and the strained atoms around the *SIA*, which have potential energy deviation from bulk value larger than 0.2 eV. The smaller spheres represent other atoms within the GB. The red circle *B* in the initial state marks the *SIA* in the final state. In (b), the energy variations on the three paths are given. Diffusion via the path *A*→*C*→*D*→*B* involves concerted exchange of three atoms, initiated by the migration of the atom *D* towards the atom *B*. The diffusion via *A*→*B* is a direct *SIA* jump from *A* to *B*. The easiest transition via *A*→*D*→*B* involves a concerted motion of two atoms.

**Fig. 9:**
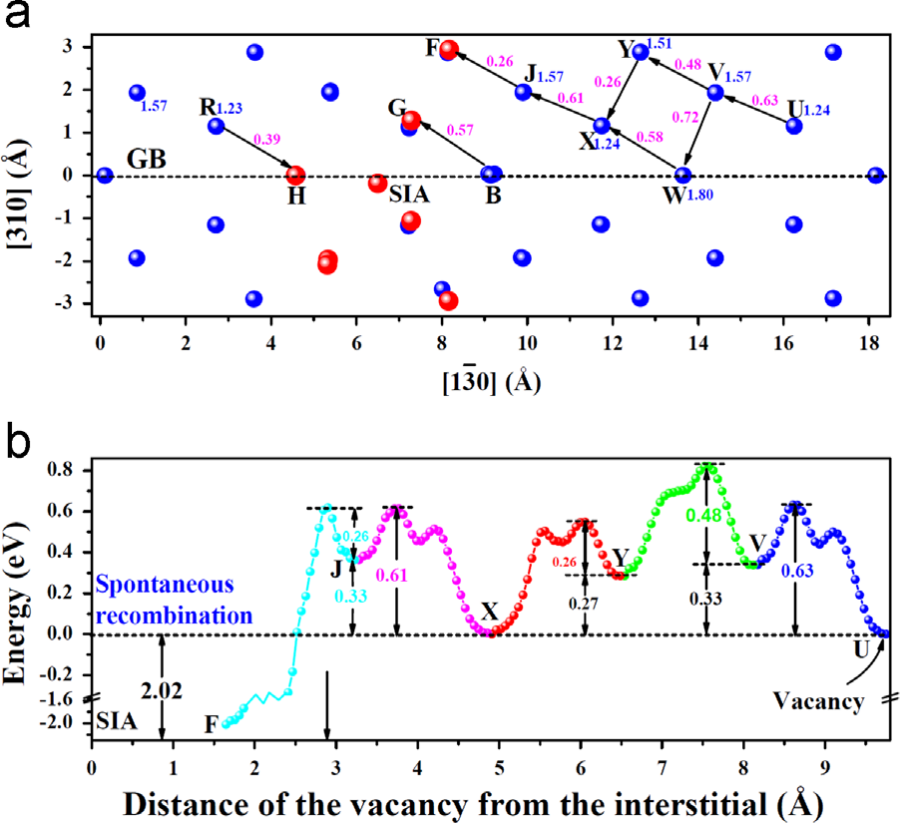
Interaction of the vacancy with the interstitial along axis [1 $\overset{¯}{3}$ 0] within the GB. In (a), the red spheres represent the sites at which one *V* spontaneously recombines with the *SIA* that locates at the GB. The blue number near the site is the *V* formation energy, while the pink number labeled on the migration path is the energy barrier. (b) shows the energy profile for the *V* migrating towards the *SIA* and then recombining with the *SIA*. The *V* initially locates at its stable site *U*. The *V* has lowest formation energy at such sites as *U* and *X*.

**Fig. 10:**
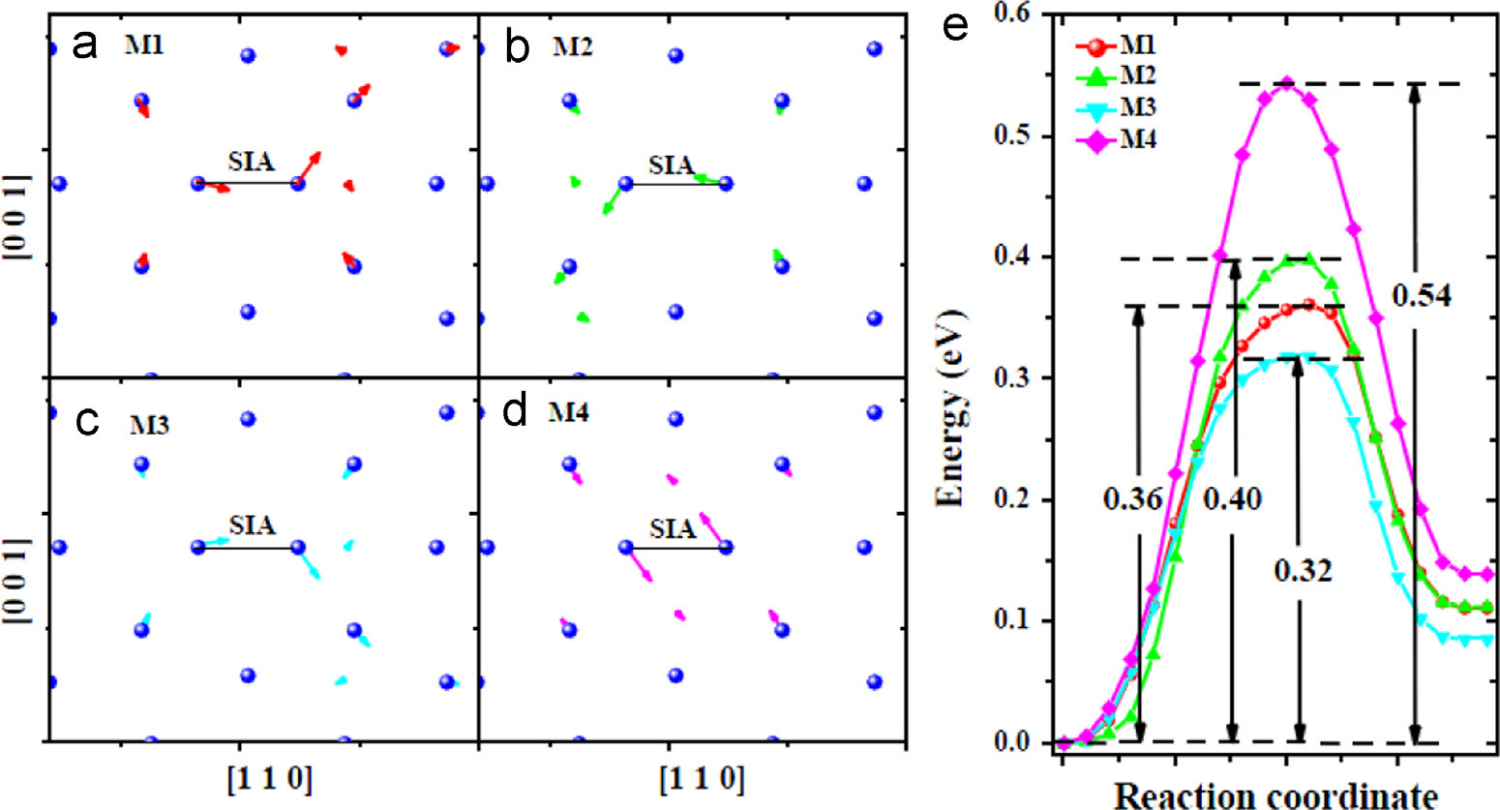
Transitions related to the *SIA* migration near one *V* in the bulk obtained via dimer [3] searches. (a–d) show the migration process visualized with the method identical to that in Fig. 4(c) and (d). (e) shows the energy profiles along the migration paths. The migration modes in (a-c) follow the Johnson׳s mechanism, i.e., the interstitial migrates via translation and rotation, while the motion in (d) corresponds to rotation of the *SIA* from one 〈1 1 0〉 to another 〈1 1 0〉.

**Fig. 11:**
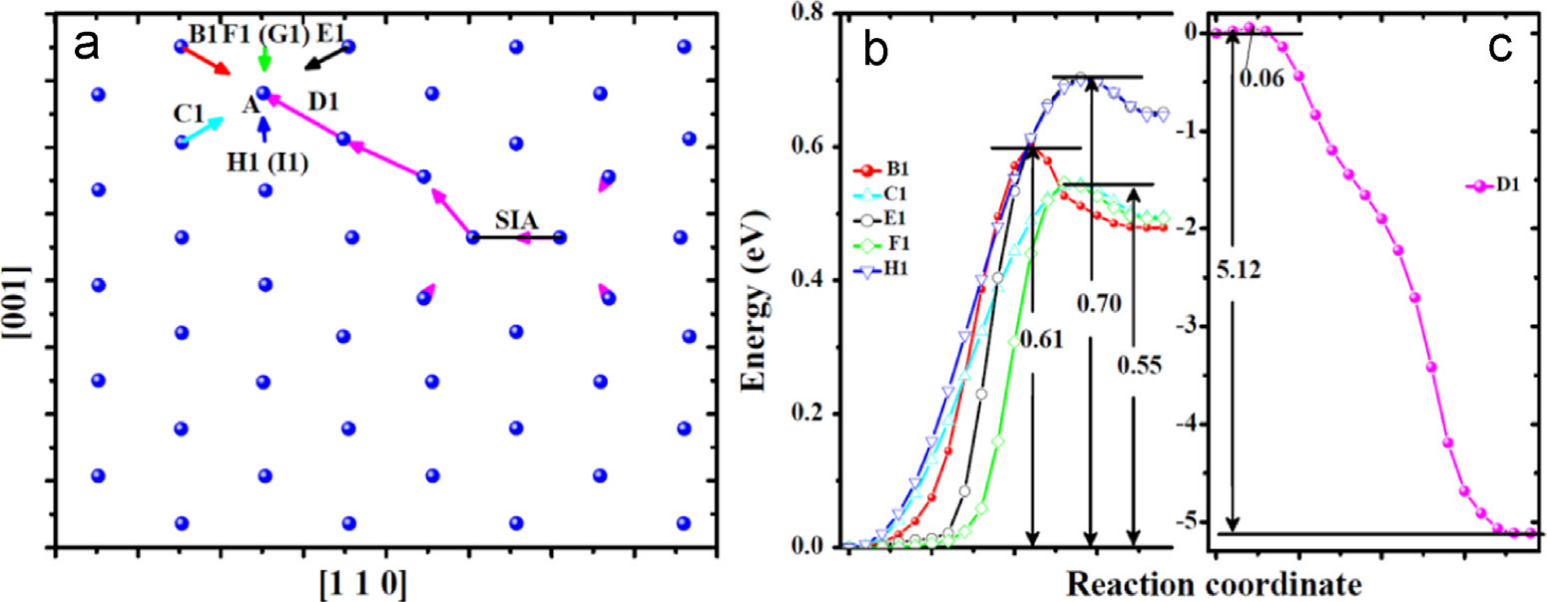
Transitions related to the migration of the *V* near one *SIA* in the bulk obtained via dimer searches. The *V* was created at site *A*. (a) shows the migration processes visualized with the same method as that in Fig. 4(c) and (d). The arrows in each mode connecting initial and final locations of the atom involved in the transition have an individual color. *B*1, *C*1, *D*1, *E*1, *F*1, *G*1, *H*1 and *I*1 mark migration modes. Modes *B*1, *C*1, *E*1, *F*1 and *H*1 correspond to the migration of the *V* from one stable site to one meta-stable site, while mode *D1* actually corresponds to the annihilation of the *V* with the *SIA*. The energy profiles for these transitions are shown in (b) and (c). Note that, the profile for *F*1 overlaps with that for *G*1, and the profile for *H*1 also overlaps with that for *I*1.

**Fig. 12:**
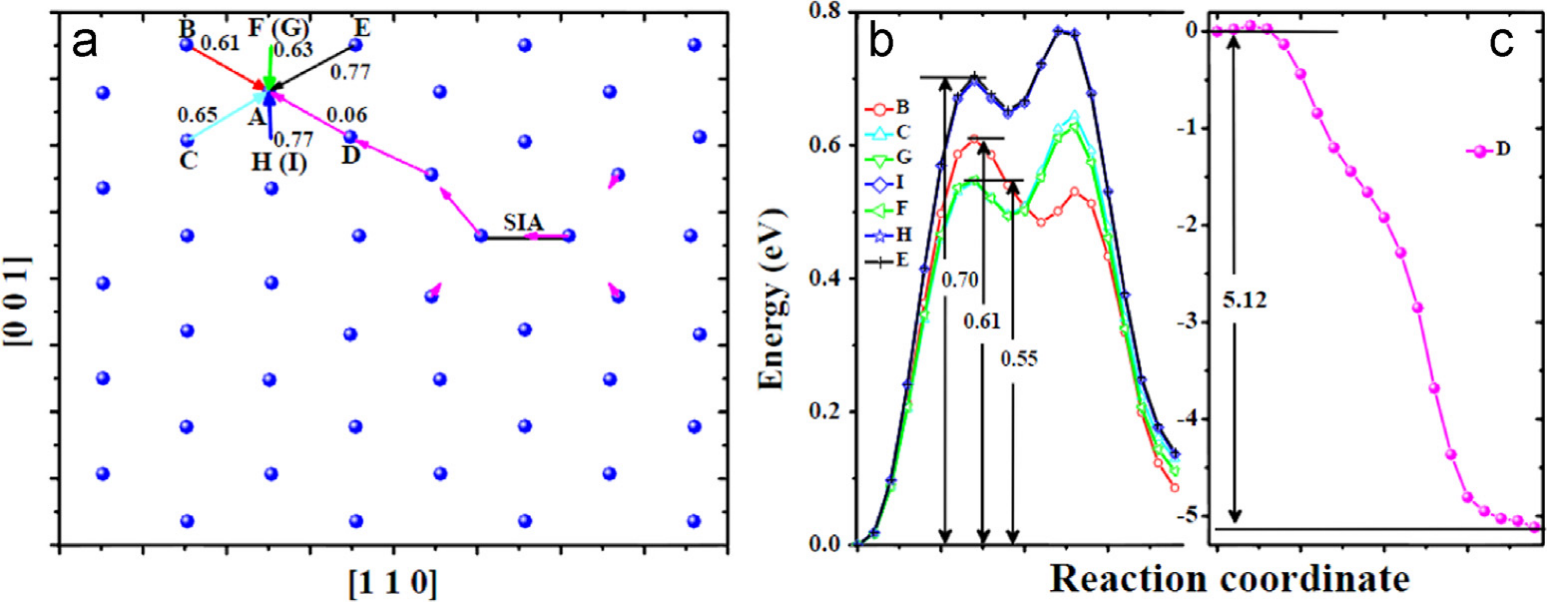
Migration of the *V* near one *SIA* in the bulk obtained via molecular statics calculations at 0 K. The *V* was created at site *A*. (a) shows the migration processes visualized with the same method as that in Fig. 4(c) and (d). The arrows in each mode connecting initial and final locations of the atom involved in the transition have an individual color. *B*, *C*, *D*, *E*, *F*, *G*, *H* and *I* mark migration modes which were artificially designed based on simple migration of the *V*, i.e., exchange with its nearest atom. Modes *B*, *C*, *E*, *F*, *G*, *H* and *I* correspond to the migration of the *V* from one stable site to another, while mode *D* corresponds to the annihilation of the *V* with the *SIA*. The energy profiles for these transitions are shown in (b) and (c).

**Fig. 13:**
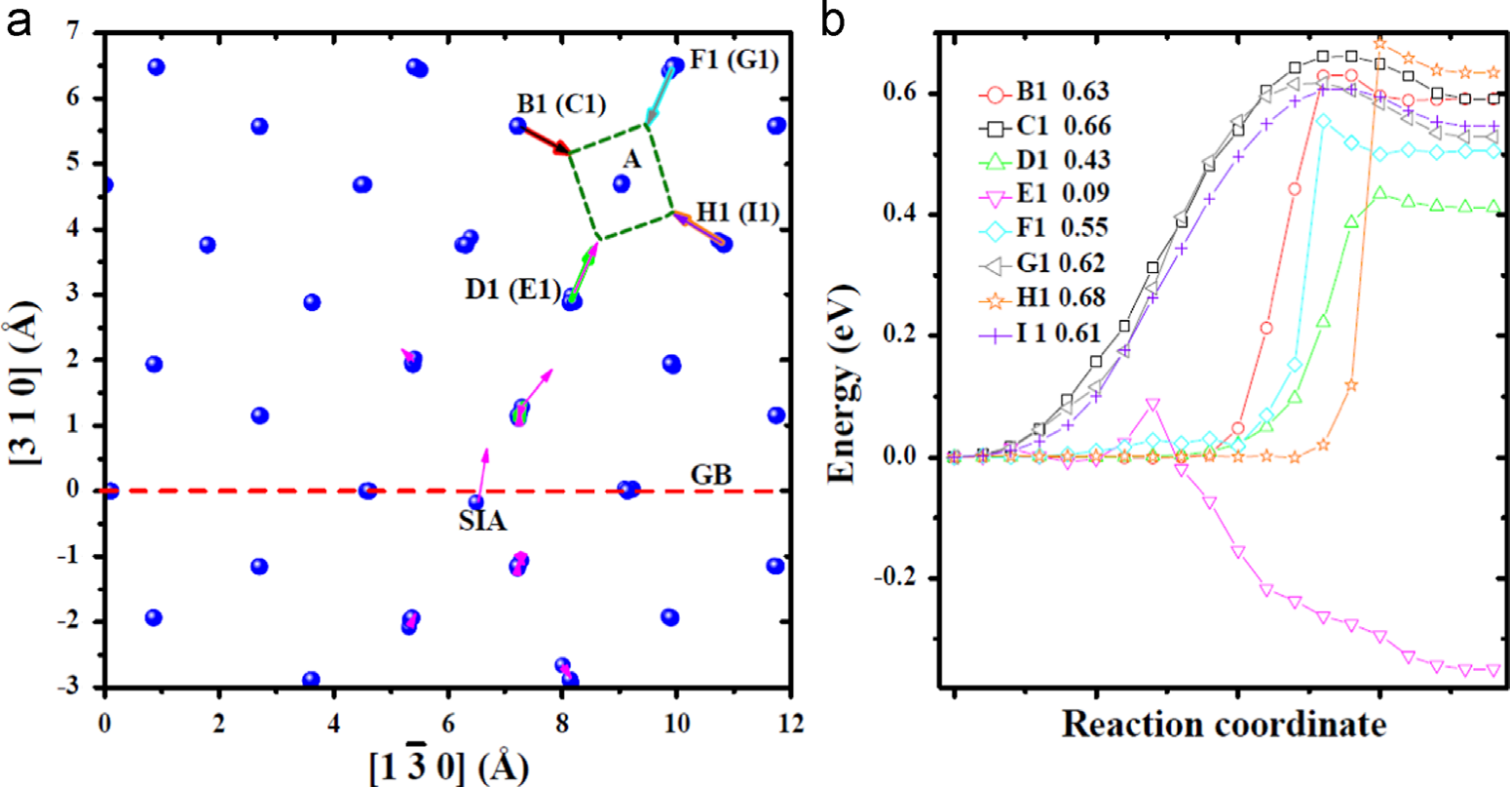
Transitions related to the migration of the *V* near one *SIA* at the GB obtained via dimer searches. The *V* was created at site *A*. (a) shows the migration processes visualized with the same method as that in Fig. 4(c) and (d). The arrows in each mode connecting initial and final locations of the atom involved in the transition have an individual color. *B*1, *C*1, *D*1, *E*1, *F*1, *G*1, *H*1 and *I*1 mark migration modes. Modes *B*1, *C*1, *D*1, *F*1 and *H*1 correspond to the migration of the *V* from one stable site to one meta-stable site, while mode *D*1 may correspond to the mediate state of the annihilation of the *V* with the *SIA*. The GB position is indicated by one red dashed line. The energy profiles for these transitions are shown in (b).

**Fig. 14:**
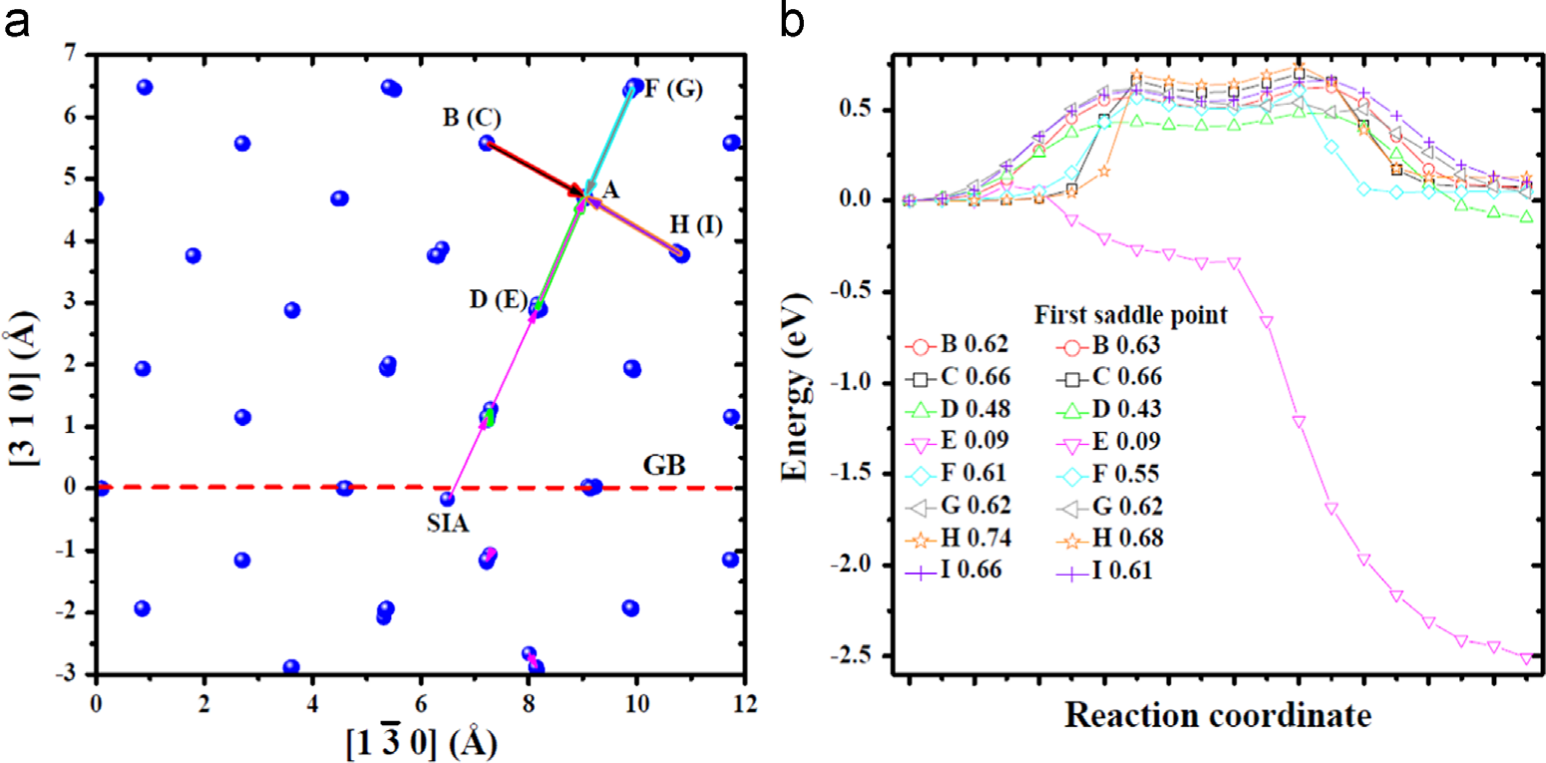
Migration of the *V* near one *SIA* at the GB obtained via molecular statics calculations at 0 K. The *V* was created at site *A*. (a) shows the migration processes visualized with the same method as that in Fig. 4(c) and (d). The arrows in each mode connecting initial and final locations of the atom involved in the transition have an individual color. *B*, *C*, *D*, *E*, *F*, *G*, *H* and *I* mark migration modes which were artificially designed based on simple migration of the *V*, i.e., exchange with its nearest atom. Modes *B*, *C*, *D*, *F*, *G*, *H* and *I* correspond to the migration of the *V* from one stable site to another, while mode E corresponds to the annihilation of the *V* with the *SIA*. The GB position is indicated by one red dashed line. The energy profiles for these transitions are shown in (b).

**Fig. 15:**
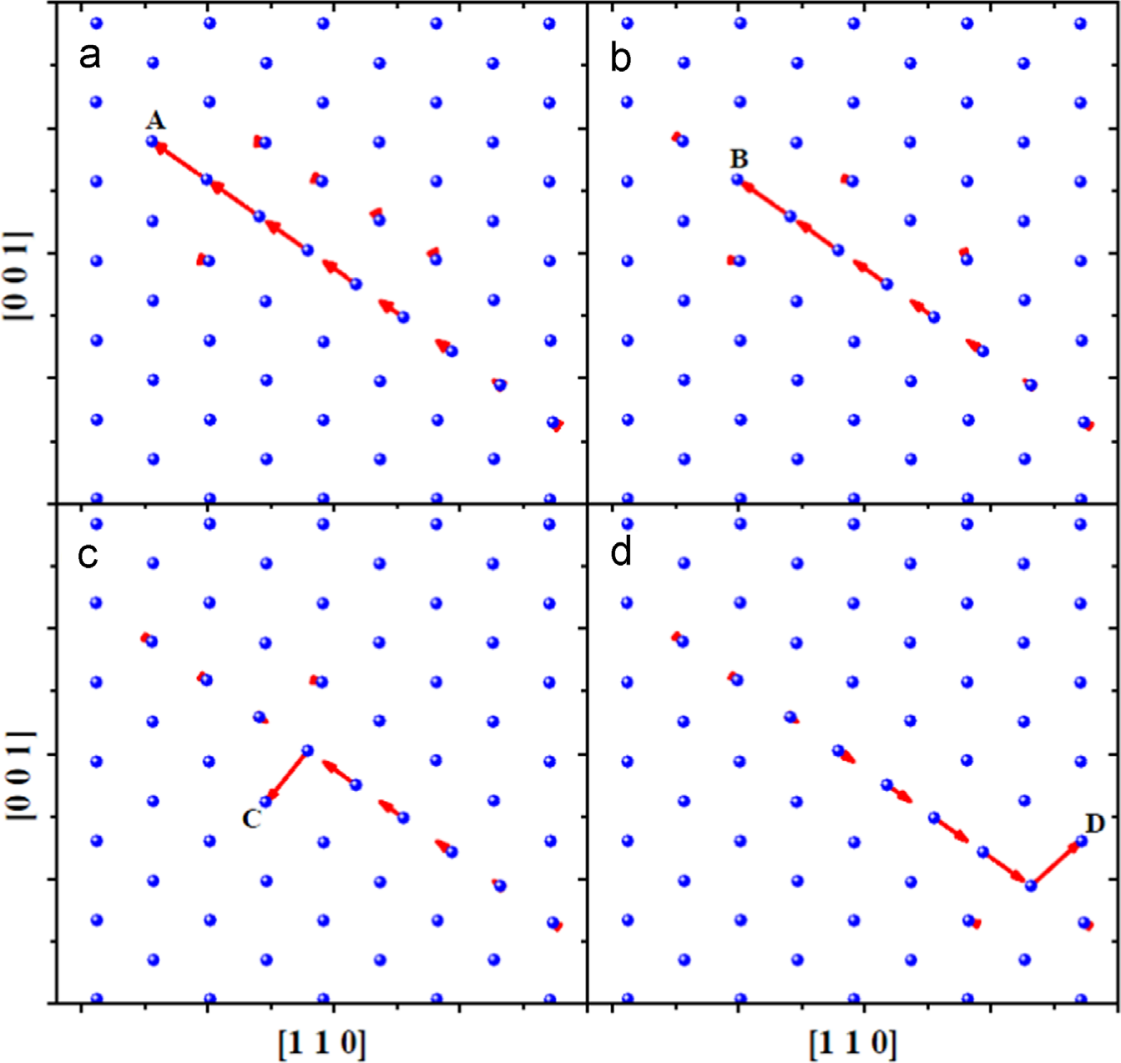
Spontaneous annihilation of one *V* with one *SIA* in tungsten bulk. The vacancy was created at sites *A*, *B*, *C* and *D*, respectively in (a–d). The visualization method for the annihilation process is the same as that in Fig. 4(c) and (d).

**Fig. 16:**
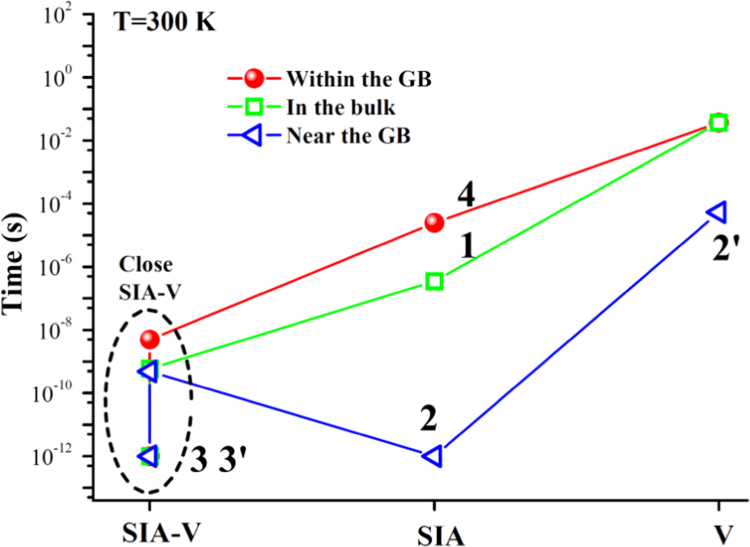
The transition time of isolated *SIA*s and vacancies diffusion, and close *SIA*-*V* pair annihilation at room temperature. The transition time is calculated via $t=t_{0}\exp(E_{a}/k_{B}T)$, where $t_{0}$ is the vibration period of atoms in iron bulk and is generally assumed to be $10^{-12}$s. The Boltzmann constant *k*_*B*_ has a value of 8.617×10^−5^ eV/K. The temperature *T* is 300 K. The relevant barriers *E*_*a*_ are given in Table 1. The numbers 1, 2, 2ʹ, 3, 3ʹ, 4 label the fundamental atomic processes near the GB, as shown in Fig. 2c in Ref. [1].

**Fig. 17:**
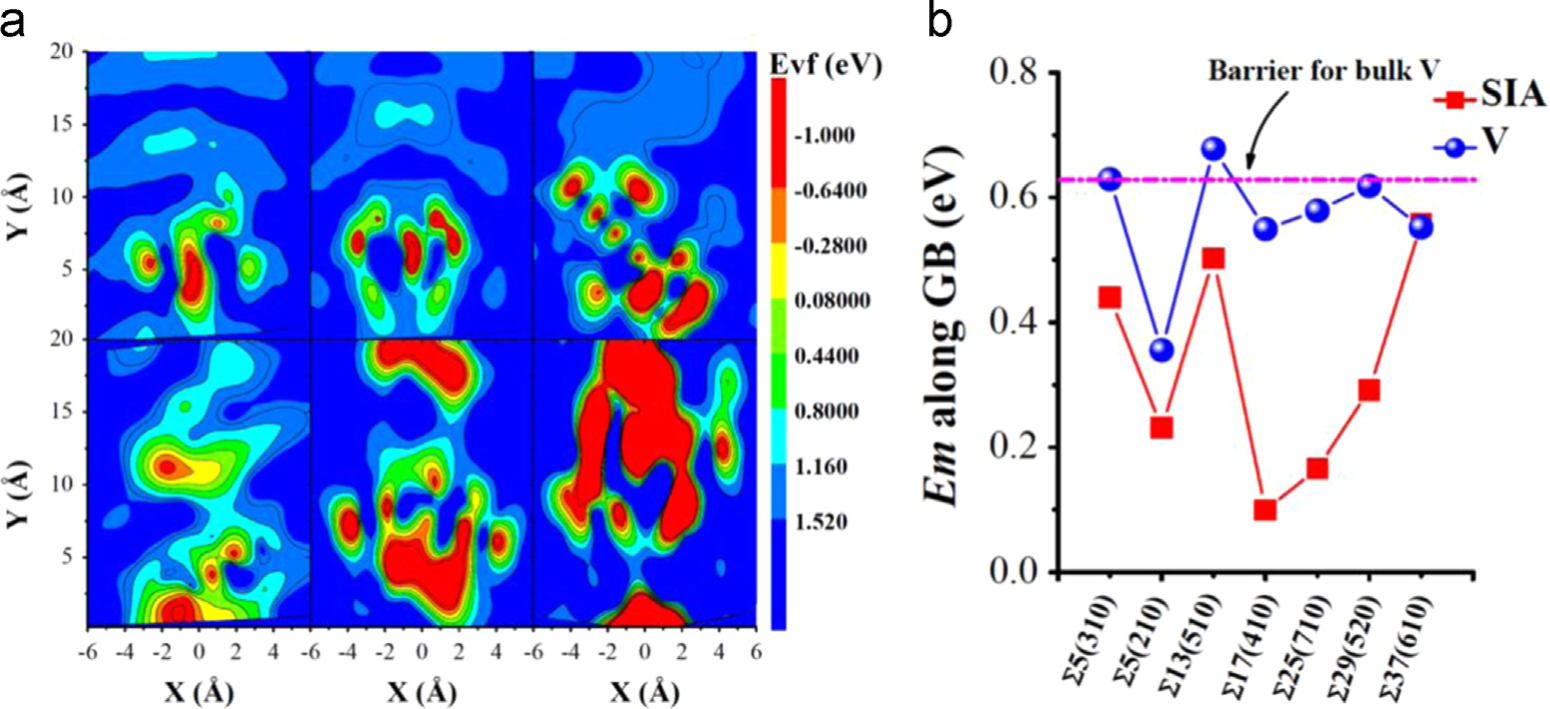
Vacancy formation energy (*E*_*vf*_) around one interstitial at the GB and the diffusion energy barriers for the single *SIA* and *V* along the GB. In (a), *E*_*vf*_ of the sites near the GB with one *SIA* trapped at the GB is shown. The sites are colored by the *E*_*vf*_ at the site. The figures in the first row are for the GBs ∑5 (2 1 0), ∑13 (5 1 0) and ∑17 (4 1 0). The axis *X* is along [2 1 0], [5 1 0] and [4 1 0], respectively. The axis *Y* is along [1 $\bar{2}$ 0], [1 $\bar{5}$ 0] and [1 $\bar{4}$ 0], respectively. The figures in the second row are for the GBs ∑25 (7 1 0), ∑29 (5 2 0) and ∑37 (6 1 0). In this case, the axis *X* is along [7 1 0], [5 2 0] and [6 1 0], respectively. The axis *Y* is along [1 $\bar{7}$ 0], [2 $\bar{5}$ 0] and [1 $\bar{6}$ 0], respectively. The GB is at the zero point on the axis *X* and parallel to the axis *Y*. In (b), the energy barriers for *SIA* and *V* diffusion along another six GBs are provided besides for that along the GB ∑5 (3 1 0).

**Fig. 18:**
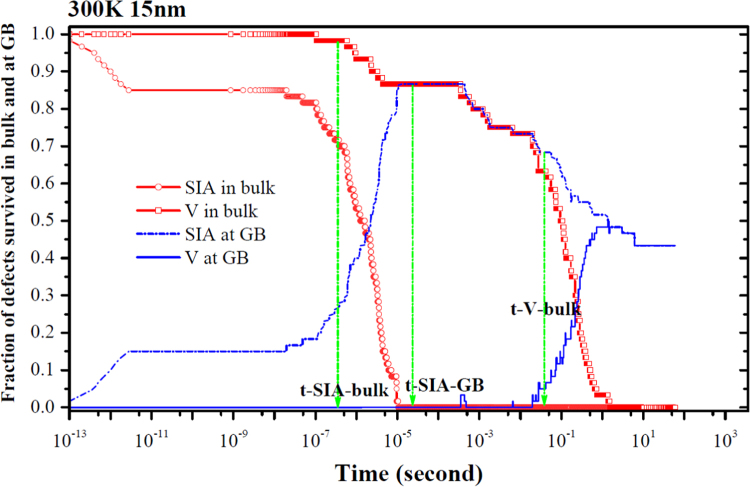
Evolution of the fraction of interstitials and vacancies in the bulk region and at the GB as a function of time at room temperature. Here, *t-SIA-bulk*, *t-SIA-GB* and *t-V-bulk* denote the transition times for the *SIA* diffusion in the bulk and within the GB and for the *V* diffusion in the bulk region. Similar to that in Fig. 1 where only segregation events are incorporated in the model, the GB effect is also seen.

**Fig. 19:**
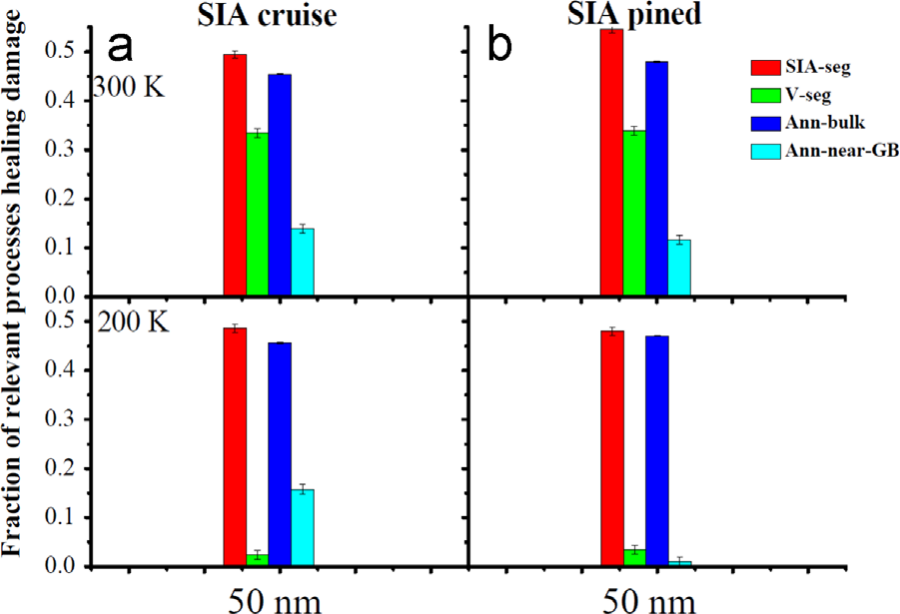
The fraction of the defects segregating into the GB and annihilated in the bulk and near the GB during annealing of the defect structure at two typical temperatures of 300 K and 200 K. In (a), after the *SIA*s get trapped by the GB, they can migrate along the GB. In (b), the *SIA*s are artificially pined at the GB. Here, the symbols *SIA-seg*, *V-seg*, and *Ann-bulk* and *Ann-GB* are short for segregation of the *SIA* and the *V*, and annihilation of the *SIA*-*V* pair in the bulk and near the GB, respectively. The initial concentration of the *SIA* and *V* is 100 appm. Each point is averaged over 8 independent OKMC runs.

**Table 1:** Kinetic parameters about *V*, *SIA* diffusion and their annihilation in the bulk region, near the GB and within the GB. These include the diffusion barrier for the *V* and *SIA* as they are far away from each other, and the recombination barrier for a close *SIA*-*V* pair located in the bulk, near the GB and within the GB. The corresponding activation temperature (*T*_*a*_) is also given, defined as the temperature that gives the transition time one second. The transition time$t=t_{0}\exp(E_{a}/k_{B}T)$, where $t_{0}$ is the vibration period of atoms in iron bulk and is generally assumed to be $10^{-12}$s. The Boltzmann constant *k*_*B*_ has a value of 8.617×$10^{-5}$ eV/K. *E*_*a*_ and *T* are activation energy and system temperature, respectively. *R* is the interaction range for an *SIA*-*V* pair or for one *V*/*SIA* with the GB. Here *V* (*SIA*) is short for the *V* (*SIA*). Other symbols are defined as follows. $ann_{SIA-V}^{sr}$: *SIA*-*V* annihilation within the spontaneous recombination region; $ann_{SIA-V}^{cp}$: *SIA*-*V* annihilation near the spontaneous annihilation region. $SIA_{m}$: diffusion for the *SIA*; $V_{m}$: diffusion for the *V*; $SIA_{escape}$: migration out of the GB for the *SIA*; $V_{escape}$: migration out of the GB for the *V*. Note that, within the GB, two parameters of *E*_*a*_/*T*_*a*_ correspond to the activation energy/temperature along two directions for the *SIA* and *V* migration.

	In the bulk region			Near the GB			Within the GB				
	*E*_*a*_ (eV)	*T*_*a*_ (K)	*R* (nm)	*E*_*a*_ (eV)	*T*_*a*_ (K)	*R* (nm)	*E*_*a*_ (eV)	*T*_*a*_ (K)	*E*_*a*_ (eV)	*T*_*a*_ (K)	*R* (nm)
$ann_{SIA-V}^{sr}$	0.00	0	0.50	0.00	0	0.28	0.00	0			0.15
$ann_{SIA-V}^{cp}$	0.17	71	0.82	0.23	97	0.53	0.22	92			0.29
$SIA_{m}$	0.33	139		0.00	0	0.83	0.44	185	1.29	542	
$V_{m}$	0.63	265		0.46	193	0.56	0.63	265	0.81	340	
$SIA_{escape}$				3.03	1273	0.83					
$V_{escape}$				1.11	466	0.56					

**Table 2:** Comparison of the interaction between an *SIA* and one *V* in Fe, W and Cu.

	Fe [1]	W [4]	Cu [5]
Effect of *SIA* on *V*	Reduced *E*_*vf*_ and *E*_*ann*_	Reduced *E*_*vf*_ and *E*_*ann*_	Reduced *E*_*vf*_ and *E*_*ann*_
Site stability	Negative *E*_*vf*_; Spontaneous annihilation region	Negative *E*_*vf*_; Spontaneous annihilation region	Negative *E*_*vf*_; Spontaneous annihilation region
Annihilation process	Replacement process; 〈1 1 1〉+〈1 1 0〉	Replacement process; 〈1 1 1〉	Replacement process; 〈1 1 0〉
Mutual motion of *SIA* and *V*; Concerted motion of a chain of atoms towards *V*	Mutual motion of *SIA* and *V*; Concerted motion of a chain of atoms towards *V*	*V* remains immobile and *SIA* is emitted
Visualization method	Connect initial and final states; monitor atom displacements variation with time	Connect initial and final states; monitor atom displacements variation with time	Connect initial and final states
