# Supplementary material for: Energetic and kinetic dataset on interaction of the vacancy and self-interstitial atom with the grain boundary in α-iron
Source: Data Brief. 2016 Mar 19;7:798–813. doi: 10.1016/j.dib.2016.03.052 (PMC4816880; doi:10.1016/j.dib.2016.03.052)
Supplement: Supplementary file 2 — Supplementary material [file mmc1.docx]

We declare that we have no financial and personal relationships with other people or organizations that can inappropriately influence our work; there is no professional or other personal interest of any nature or kind in any product, service and / or company that could be construed as influencing the position presented in or the review of the manuscript entitled “Energetic and kinetic dataset on interaction of the vacancy and self-interstitial atom with the grain boundary in α-iron”.

**Authors:**

Xiangyan Li^1^, Wei Liu^1^, Yichun Xu^1*^, C.S. Liu^1*^, B.C. Pan^2^, Yunfeng Liang^1,3^, Q.F. Fang^1^, Jun-Ling Chen^4^, G.-N. Luo^4^, Guang-Hong Lu^5^, Zhiguang Wang^6^

**Affiliations:**

^1^Key Laboratory of Materials Physics, Institute of Solid State Physics, Chinese Academy of Sciences, P.O. Box 1129, Hefei 230031, PR China

^2^Hefei National Laboratory for Physical Sciences at Microscale and Department of Physics, University of Science and Technology of China, Hefei 230026, PR China

^3^Environment and Resource System Engineering, Kyoto University, Kyoto 615-8540, Japan

^4^Institute of Plasma Physics, Chinese Academy of Sciences, Hefei 230031, PR China

^5^Department of Physics, Beihang University, Beijing 100191, PR China

^6^Institute of Modern Physics, Chinese Academy of Sciences, Lanzhou 730000, PR China
